# Retrospective cohort study of an MPEWS-triggered personalized care bundle for critically ill children in an emergency observation unit

**DOI:** 10.1097/MD.0000000000047042

**Published:** 2026-01-23

**Authors:** Yafei Liu, Xuejuan He, Lifeng Wei, Hui Hu, Yilin Miao, Lingfang Yu, Yuqian Weng

**Affiliations:** a Department of Pediatric Emergency Care Area, The Affiliated Yangming Hospital of Ningbo University, Yuyao City, Zhejiang Province China; b Department of Outpatient, The Affiliated Yangming Hospital of Ningbo University, Yuyao City, Zhejiang Province, China.

**Keywords:** critically ill children, emergency observation, modified pediatric early warning score, personalized care

## Abstract

This study aimed to evaluate whether a modified pediatric early warning score (MPEWS)-triggered personalized care bundle – designed to facilitate early detection of clinical deterioration and guide timely individualized interventions – improves outcomes in critically ill children in an emergency observation unit. A retrospective cohort study was conducted among 120 critically ill children admitted to the emergency observation unit. Based on the care received, patients were allocated to either the MPEWS-based personalized care group (n = 60) or the routine care group (n = 60). Data were extracted from electronic medical records. Primary outcomes included stabilization of vital signs, length of stay, incidence of complications, and parent-reported satisfaction. Children in the MPEWS group achieved significantly faster stabilization of heart rate, respiratory rate, blood pressure, oxygen saturation, and consciousness level within 24 to 72 hours compared with those receiving routine care (*P* < .05). The median length of stay was shorter in the MPEWS group (3.0 days [interquartile range: 2.0–4.0] vs 4.0 days [interquartile range: 3.0–5.0], *P* < .001). Complication rates were lower, including respiratory failure (6.7% vs 20.0%), cardiac arrest (0% vs 6.7%), and hospital-acquired infections (5.0% vs 16.7%; *P* < .05). Parent satisfaction was also higher in the MPEWS group (median 98 [95–100] vs 90 [85–94], *P* < .001). MPEWS-triggered personalized care was associated with more rapid physiological stabilization, reduced hospitalization time, fewer complications, and greater family satisfaction in critically ill children in emergency observation settings.

## 1. Introduction

Critically ill children in emergency observation units (EOUs) require prompt, effective interventions to optimize outcomes and prevent deterioration. While early recognition and management of clinical deterioration are vital for reducing morbidity and mortality, traditional routine care often lacks the individualization and timeliness needed for these complex cases.

The modified pediatric early warning score (MPEWS) offers a validated tool for assessing illness severity and predicting deterioration risk in pediatric patients.^[1,2]^ Based on heart rate, respiratory rate, systolic blood pressure, temperature, and consciousness level. MPEWS provides an objective, standardized measure of clinical status. Research demonstrates its effectiveness in identifying at-risk children and guiding care escalation across emergency departments, inpatient wards, and intensive care units.^[3-5]^

Personalized care interventions, tailored to individual clinical conditions, developmental stages, and psychosocial factors, can enhance outcomes and patient satisfaction.^[6]^ Integrating MPEWS into these interventions may further optimize care delivery for critically ill children in EOUs.

While studies have examined MPEWS in pediatric emergency care,^[7,8]^ evidence for MPEWS-based personalized interventions in EOUs remains limited. Agulnik et al^[9]^ found that implementing a pediatric early warning system reduced clinical deterioration (relative risk = 0.38) and pediatric intensive care unit (PICU) transfer needs (relative risk = 0.26) in pediatric oncology patients. Mandell et al^[10]^ demonstrated that higher PEWS scores at PICU discharge correlated with increased unplanned readmission risk within 48 hours (odds ratio = 1.50 per 1-point increase).

Despite these findings, high-quality randomized controlled trials (RCTs) evaluating MPEWS-based personalized care interventions in EOUs are lacking. This study addresses this gap through an RCT investigating the impact of MPEWS-based personalized care on critically ill children’s outcomes. We hypothesize that this approach, compared to routine care, will improve clinical conditions, stabilize vital signs, reduce length of stay and complications, and enhance patient satisfaction.

## 2. Materials and methods

### 2.1. Study design and setting

This study was designed as a single-center, retrospective cohort study and was conducted in the 20-bed EOU of Yuyao People’s Hospital, a tertiary care children’s hospital in China. Clinical data were retrieved from electronic medical records of critically ill children admitted to the EOU between January 1 and December 31, 2022. Each patient was followed from the time of admission to the EOU until discharge or transfer to the PICU, with a maximum observation period of 72 hours, in accordance with the unit’s clinical protocols. The EOU provides short-term intensive monitoring and stabilization for children who require close observation but do not initially meet PICU admission criteria, such as the need for prolonged mechanical ventilation, continuous vasoactive infusion, or multi-organ support. Patients who deteriorated clinically or required advanced life support were promptly transferred to the PICU according to hospital policy. The study was approved by the Ethics Committee of Yuyao People’s Hospital. Owing to its retrospective design and use of anonymized data, informed consent was waived in accordance with institutional and national ethical guidelines.

### 2.2. Participants

Eligibility criteria included: age ≤ 14 years, critical illness requiring immediate intervention, expected stay ≥ 24 hours, and MPEWS score ≥ 5 at admission.^[11]^ Exclusion criteria were: expected death within 24 hours, planned transfer/discharge within 24 hours, or refused consent. Sample size was calculated based on the primary endpoint of time-to-discharge, assuming a hazard ratio of 1.6 for earlier discharge in the intervention group compared to control, with α = 0.05, 90% power, and 10% attrition. This required 60 participants per group.^[12]^

### 2.3. Grouping and bias mitigation

Patients were assigned to exposure groups based on the actual care received during their EOU stay, as documented in the electronic medical record. Children who received the MPEWS-triggered personalized care bundle were classified as the MPEWS group, whereas those managed according to standard unit protocols were classified as the routine care group. No randomization or allocation concealment was performed, consistent with the retrospective cohort design. To limit selection and information bias, we applied the following measures: consecutive case identification within the prespecified study window; predefined inclusion/exclusion criteria and a standardized data-abstraction form; and chart reviewers and outcome assessors who were masked to group labels during data extraction and adjudication of complications. Because blinding of bedside clinicians and families is not applicable in a retrospective design, performance bias cannot be fully excluded.

Potential confounding was addressed by adjusting for clinically relevant baseline covariates (age, sex, baseline MPEWS, PRISM III score, primary diagnosis, and key comorbidities) in multivariable analyses (see Section 2.7).

### 2.4. Intervention

Control group (routine care): children in the control group received standard care accordings to our hospital’s EOU protocols. This included:

Respiratory support: supplemental oxygen if SpO_2_ < 92% or as clinically indicated. noninvasive ventilation or high-flow nasal cannula was initiated for moderate respiratory distress, while invasive mechanical ventilation was escalated if clinical deterioration continued or if the child met standard PICU transfer criteria.

Cardiovascular management: establishment of at least 1 peripheral intravenous line for fluid resuscitation (e.g., isotonic crystalloid boluses in cases of hypovolemia). Vasoactive medications (e.g., dopamine, dobutamine) were administered only if strictly necessary, typically after consultation with critical care and based on standardized protocols.

Medication: antibiotic therapy was empirical, guided by local microbiological patterns, and adjusted upon culture results. Diuretics were prescribed for fluid overload states or congestive heart failure. Analgesics and antipyretics were used as needed.

Intervention group (MPEWS-based personalized care): In addition to routine care, children in the intervention group were managed using an MPEWS-triggered care model. In this study, MPEWS was not used solely as a monitoring or assessment tool; rather, it served a dual function: as a structured scoring system for the early identification of clinical deterioration and as a clinical trigger to initiate predefined interventions and activate the personalized care bundle. Accordingly, changes in MPEWS scores and threshold values directly guided decisions regarding escalation of respiratory support, activation of the rapid response team (RRT), medication adjustments, and targeted nursing measures. This included:

Frequent MPEWS assessments: assessment of the child’s condition using MPEWS every 1 to 2 hours. The MPEWS was calculated based on the child’s heart rate, respiratory rate, systolic blood pressure, temperature, and level of consciousness, according to the age-specific criteria shown in Table 1.^[13]^ A MPEWS score of 0 to 2 points indicated a low risk of clinical deterioration, 3 to 4 points indicated a medium risk, and ≥5 points indicated a high risk.

**Table 1:** The modified pediatric early warning score (MPEWS) used in this study.

Parameters	Scores				
	0	1	2	3	4
Heart rate (beats/min)					
<1 yr	90–160	80–89 or 161–180	70–79 or 181–190	60–69 or 191–200	<60 or >200
1–4 yr	80–140	70–79 or 141–160	60–69 or 161–170	50–59 or 171–180	<50 or >180
5–12 yr	70–120	60–69 or 121–140	50–59 or 141–150	40–49 or 151–160	<40 or >160
>12 yr	60–100	50–59 or 101–120	40–49 or 121–130	30–39 or 131–140	<30 or >140
Respiratory rate (breaths/min)					
<1 yr	30–50	20–29 or 51–60	15–19 or 61–70	10–14 or 71–80	<10 or >80
1–4 yr	20–40	15–19 or 41–50	10–14 or 51–60	5–9 or 61–70	<5 or >70
5–12 yr	15–30	10–14 or 31–40	5–9 or 41–50	3–4 or 51–60	<3 or >60
>12 yr	10–20	8–9 or 21–30	6–7 or 31–40	4–5 or 41–50	<4 or >50
Systolic blood pressure (mm Hg)					
<1 mo	60–90	50–59 or 91–100	40–49 or 101–110	30–39 or 111–120	<30 or >120
1–12 mo	70–100	60–69 or 101–110	50–59 or 111–120	40–49 or 121–130	<40 or >130
1–4 yr	80–110	70–79 or 111–120	60–69 or 121–130	50–59 or 131–140	<50 or >140
5–12 yr	90–120	80–89 or 121–130	70–79 or 131–140	60–69 or 141–150	<60 or >150
>12 yr	100–130	90–99 or 131–140	80–89 or 141–150	70–79 or 151–160	<70 or >160
Temperature (°C)	36.0–38.0	35.0–35.9 or 38.1–39.0	34.0–34.9 or 39.1–40.0	33.0–33.9 or 40.1–41.0	<33.0 or >41.0
Consciousness level	Alert	Voice	Pain	Unresponsive	

MPEWS = modified pediatric early warning score.

Rapid response team involvement: for sustained high MPEWS or clinical worsening, the RRT (pediatric emergency physician, pediatric critical care physician, senior pediatric nurse) performed advanced interventions such as early noninvasive or invasive ventilation, continuous vasoactive support (if indicated by shock states), and expedited imaging or procedures. Targeted nursing interventions (e.g., meticulous airway clearance, pressure ulcer prevention, age-appropriate coping strategies) tailored to the child’s evolving clinical status and preferences of the child and family. Medication adjustments: Antibiotics, diuretics, and other medications were reviewed more frequently to ensure early optimization based on clinical and laboratory parameters. Ongoing team education and feedback: nursing staff received additional training sessions, while daily huddles and case reviews were employed to reinforce the personalized, MPEWS-driven approach.

Children in the intervention group received an MPEWS-triggered care bundle. This bundle consisted of: structured monitoring cadence with MPEWS assessment every 1 to 2 hours, predefined thresholds for RRT activation (MPEWS ≥ 5 or sustained worsening), targeted nursing actions (airway clearance, pressure ulcer prevention, psychosocial support), structured medication reviews every 12 to 24 hours, and daily team huddles for multidisciplinary coordination. The bundle was delivered by trained pediatric nurses, emergency physicians, and critical care physicians. Tailoring was based on evolving clinical status, with minor modifications (e.g., adjusted huddle timing) permitted under clinical urgency.

### 2.5. Outcome measures

The primary outcome was time-to-discharge from the EOU. Secondary outcomes included: changes in vital signs, incidence of complications, and patient satisfaction. Changes in vital signs (heart rate, respiratory rate, blood pressure, oxygen saturation, and consciousness level) from admission to 72 hours after admission, which reflected the effectiveness of the intervention in stabilizing the child’s physiological parameters. Length of stay in the EOU, which reflected the effectiveness of the intervention in facilitating the child’s recovery and discharge. Incidence of complications during the stay in the EOU, including respiratory failure requiring mechanical ventilation, shock requiring vasoactive medications, cardiac arrest requiring cardiopulmonary resuscitation, and hospital-acquired infections (pneumonia, urinary tract infection, and bloodstream infection), which reflected the effectiveness of the intervention in preventing or managing adverse events. Patient satisfaction with the care received in the EOU, as measured by a validated questionnaire^[14]^ completed by the parents or legal guardians at discharge, reflecting the intervention’s effectiveness in improving the child’s and family’s overall experience and satisfaction. The ESCAPE Patient Satisfaction Questionnaire consisted of 20 items covering communication with healthcare staff, perceived timeliness and quality of care, comfort of the hospital environment, and overall satisfaction. Each item was scored on a 5-point Likert scale (1 = very dissatisfied to 5 = very satisfied), with a total score range of 20 to 100, higher scores indicating greater satisfaction. The instrument was originally developed by Cross et al^[14]^ and has undergone formal validation in pediatric emergency care populations, including face validity (expert panel review), content validity (item relevance rated > 0.80 by pediatric emergency specialists), and convergent validity (correlation *r* = 0.72 with global satisfaction rating). For the present study, the questionnaire was culturally adapted and pilot-tested in 15 parents prior to data collection, showing high internal consistency (Cronbach’s α = 0.91).

Complications were operationally defined as: respiratory failure requiring mechanical ventilation, shock requiring vasoactive medications, cardiac arrest requiring cardiopulmonary resuscitation, and hospital-acquired infections (confirmed by microbiological culture). Complication data were collected using a structured audit tool developed by our hospital’s pediatric critical care committee, which underwent content validation by 3 senior pediatricians and demonstrated good inter-rater reliability (κ = 0.86) in pilot testing.

### 2.6. Data collection

Baseline characteristics included age, gender, diagnosis, comorbidities, and pediatric risk of mortality III score.^[15,16]^ Outcomes were collected at discharge from medical records and satisfaction questionnaires.

Intervention fidelity was assessed weekly. Adherence was measured by the percentage of on-time MPEWS checks, proportion of MPEWS ≥ threshold cases with timely RRT activation, and nursing checklist completion rates. Dose/exposure was assessed as the mean number of extra assessments per patient and frequency of team huddles delivered. Competency was evaluated via staff training participation, post-training quiz pass rates, and quarterly scoring audits. Contamination was monitored as the percentage of control patients inadvertently exposed to bundle elements.

### 2.7. Statistical analysis

All statistical analyses were performed using SPSS version 26.0 (IBM Corp., Armonk) and followed the intention-to-treat principle. Participants were analyzed in their original groups regardless of protocol adherence. Between-group comparisons were conducted using independent-samples *t* tests for normally distributed continuous variables, Mann–Whitney *U* tests for non-normally distributed data, and χ² tests or Fisher’s exact tests for categorical variables. All reported differences were considered statistically significant at *P* < .05. Changes in vital signs over time were assessed using repeated-measures analysis of variance, with time, group, and time × group interaction as fixed effects, followed by Bonferroni-adjusted post hoc comparisons when appropriate. Time-to-discharge was evaluated using Kaplan–Meier survival analysis, and differences between groups were tested with the log-rank test. Survival curves were plotted to illustrate discharge probability within the 72-hour observation period. Patients who were transferred to the PICU or withdrew were censored at the time of transfer or withdrawal.

## 3. Results

### 3.1. Participant characteristics

**Figure 1.**
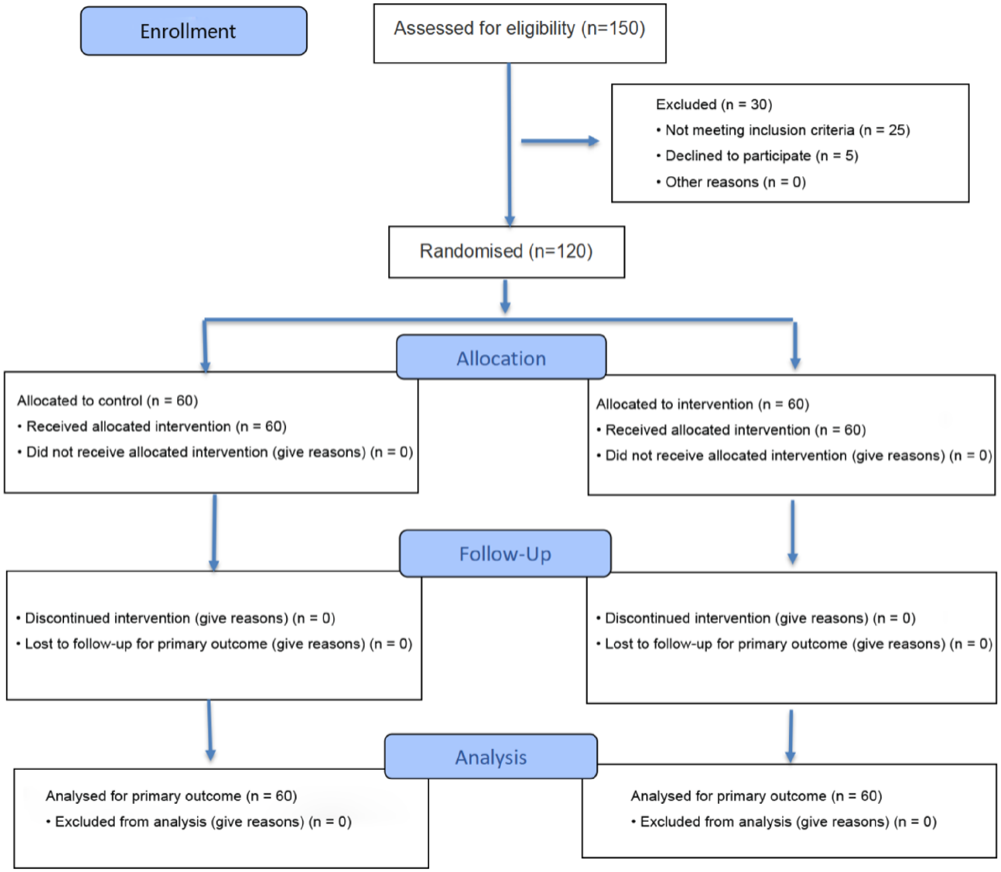
CONSORT flow diagram. CONSORT = consolidated standards of reporting trials.

The study enrolled 120 critically ill children (60 per group) with well-balanced baseline characteristics between groups (all *P* > .05) as shown in Table 2. Participants’ mean age was 5.4 ± 3.5 years, with 56.7% male. Primary diagnoses included respiratory diseases (46.7%), neurological diseases (20.0%), circulatory diseases (15.0%), digestive diseases (10.0%), and other conditions (8.3%), matching the distribution shown in Table 2. The mean pediatric risk of mortality III score was 10.8 ± 4.2, and median EOU stay was 3.5 days (interquartile range: 2.0–5.0). Comorbidities included congenital anomalies (13.3% vs 11.7%), genetic disorders (8.3% vs 10.0%), and chronic diseases (20.0% vs 16.7%), while the remainder had no comorbidities. The flow of participants through the trial is shown in Figure 1.

**Table 2:** Baseline characteristics of the participants.

Characteristic	Control group (n = 60)	Intervention group (n = 60)	*P* values
Age (yr), mean ± SD	5.3 ± 3.4	5.5 ± 3.6	.750
Male sex, n (%)	35 (58.3)	33 (55.0)	.713
Diagnosis, n (%)			.935
Respiratory diseases	28 (46.7)	26 (43.3)	
Neurological diseases	12 (20.0)	12 (20.0)	
Circulatory diseases	9 (15.0)	10 (16.7)	
Digestive diseases	6 (10.0)	7 (11.7)	
Others	5 (8.3)	5 (8.3)	
Comorbidities, n (%)			.856
Congenital anomalies	8 (13.3)	7 (11.7)	
Genetic disorders	5 (8.3)	6 (10.0)	
Chronic diseases	12 (20.0)	10 (16.7)	
None	35 (58.3)	37 (61.7)	
PRISM III score, mean ± SD	11.0 ± 4.3	10.6 ± 4.1	.612
MPEWS score at admission, median (IQR)	6 (5–7)	6 (5–7)	.845

Median emergency observation unit stay, d (IQR) – control: 4.0 (3.0–5.0); intervention: 3.0 (2.0–4.0); *P* < .001.

IQR = interquartile range, MPEWS = modified pediatric early warning score, PRISM = pediatric risk of mortality, SD = standard deviation.

### 3.2. Outcomes

**Figure 2.**
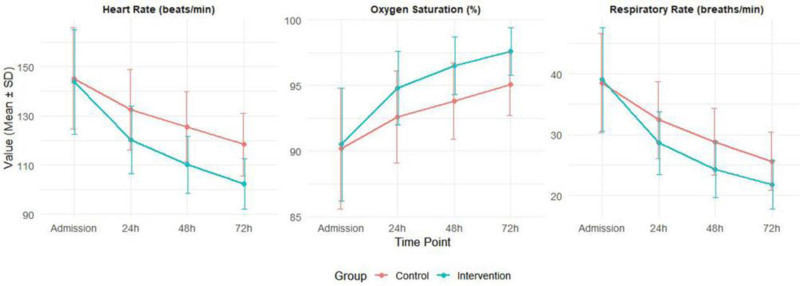
Vital sign trends over time.

Vital sign changes (Table 3) showed significantly greater stability in the intervention group at 24-, 48-, and 72-hours post-admission (all *P* < .05). Repeated-measures analysis of variances revealed significant time-group interaction effects (all *P* < .01), with both groups showing improvement from admission to 72 hours (*P* < .001), but more pronounced in the intervention group (*P* < .001). The intervention group demonstrated shorter EOU stays (median: 3.0 [2.0–4.0] vs 4.0 [3.0–5.0] days, *P* < .001). Kaplan–Meier analysis confirmed higher probability of earlier discharge in the intervention group (log-rank test: *P* < .001). Cox proportional hazards model estimated hazard ratio for discharge = 1.72 (95% confidence interval [CI]: 1.23–2.41, *P* < .001). Competing events such as PICU transfer or in-hospital death were rare; a competing-risk sensitivity analysis yielded similar results. Complication rates were lower in the intervention group: respiratory failure (6.7% vs 20.0%, *P* = .030), shock (3.3% vs 13.3%, *P* = .087), cardiac arrest (0% vs 6.7%, *P* = .042), and hospital-acquired infections (5.0% vs 16.7%, *P* = .037). Relative risks for the intervention group were: respiratory failure 0.33 (95% CI: 0.12–0.94), shock 0.25 (95% CI: 0.06–1.12), cardiac arrest 0.07 (95% CI: 0.00–1.19), and hospital-acquired infections 0.30 (95% CI: 0.09–1.00). Notably, while the point estimate suggested reduced risk of shock, the CI crossed 1.0 and the difference was not statistically significant. Patient satisfaction scores were significantly higher in the intervention group (median: 98 [95–100] vs 90 [85–94], *P* < .001), with a large effect size (Cohen’s *d* = 1.76). Figure 2 presents the trends in heart rate, respiratory rate, and oxygen saturation over the 72-hour observation period, demonstrating more rapid stabilization in the intervention group compared to the control group.

**Table 3:** Changes in vital signs over time.

Vital sign	Time point (h)	Control group	Intervention group	*P* value
Heart rate (beats/min), mean ± SD	Admission	145.2 ± 20.6	143.8 ± 21.2	.712
	24	132.5 ± 16.4	120.3 ± 13.8	<.001
	48	125.6 ± 14.1	110.2 ± 11.5	<.001
	72	118.4 ± 12.7	102.5 ± 10.2	<.001
Respiratory rate (breaths/min), mean ± SD	Admission	38.5 ± 8.2	39.1 ± 8.5	.692
	24	32.4 ± 6.3	28.6 ± 5.2	<.001
	48	28.8 ± 5.5	24.3 ± 4.6	<.001
	72	25.6 ± 4.8	21.8 ± 4.0	<.001
Systolic blood pressure (mm Hg), mean ± SD	Admission	92.5 ± 15.4	93.2 ± 14.8	.802
	24	98.6 ± 12.3	104.5 ± 10.6	.005
	48	102.4 ± 10.5	110.8 ± 9.2	<.001
	72	105.8 ± 9.6	116.2 ± 8.4	<.001
Oxygen saturation (%), mean ± SD	Admission	90.2 ± 4.6	90.5 ± 4.3	.714
	24	92.6 ± 3.5	94.8 ± 2.8	<.001
	48	93.8 ± 2.9	96.5 ± 2.2	<.001
	72	95.1 ± 2.4	97.6 ± 1.8	<.001
Consciousness level (AVPU), median (IQR)	Admission	2 (2–3)	2 (2–3)	.667
	24	2 (1–2)	1 (1–1)	<.001
	48	1 (1–2)	1 (1–1)	<.001
	72	1 (1–1)	1 (1–1)	.011

AVPU = alert, voice, pain, unresponsive, IQR = interquartile range, SD = standard deviation.

## 4. Discussion

This study demonstrates that MPEWS-based personalized care significantly improves outcomes for critically ill children in EOUs. The intervention effectively reduced illness severity, stabilized vital signs, shortened length of stay, decreased complications, and enhanced patient satisfaction compared to routine care. These findings establish MPEWS as a valuable tool for guiding individualized pediatric emergency care. While MPEWS has been validated across various pediatric settings, few studies have evaluated MPEWS-based interventions’ effectiveness in EOUs. Our study addresses this gap by demonstrating clear benefits of personalized care integration.

The personalized care intervention in this study was designed to optimize the care process and outcomes of critically ill children by integrating the MPEWS into the clinical decision-making and nursing practice. The intervention emphasized the importance of frequent and accurate assessment of the child’s condition using the MPEWS, which allowed for the early detection of deterioration and prompt initiation of appropriate interventions. The escalation of care based on the MPEWS score and clinical judgment ensured that the children received timely and adequate support from the healthcare team, including the rapid response team for those with a high risk of deterioration. The targeted nursing interventions based on the child’s specific needs addressed the multidimensional aspects of care and promoted the child’s physical, psychological, and social well-being. Protocolized bundle components, structured education, feedback, and teamwork enhanced nurses’ competency and collaboration, maintaining high-quality care throughout the study period.^[16]^

The positive effects of the personalized care intervention on the child’s outcomes can be explained by several mechanisms. First, the early recognition and treatment of deterioration by using the MPEWS may have prevented the progression of critical illness and reduced the severity and duration of organ dysfunction.^[17]^ Second, the individualized nursing interventions may have alleviated the child’s discomfort and distress, facilitated the child’s recovery and coping, and prevented the complications associated with immobility and invasive procedures.^[18]^ Third, the involvement of the family in the care process may have improved the communication and trust between the healthcare team and the family, facilitated the family’s understanding and participation in the child’s care, and enhanced the family’s satisfaction with the care received.^[19]^ Fourth, the multidisciplinary collaboration and quality improvement may have optimized the coordination and continuity of care, minimized the errors and delays in the care process, and ensured adherence to the best practice guidelines.^[20,21]^

Study strengths include robust RCT design with comprehensive outcome measures, rigorous intervention protocol, and appropriate statistical analysis. However, limitations exist. First, this was a single-center study, limiting generalizability. Second, the short observation horizon (≤72 hour) restricted assessment of long-term outcomes. Third, there was a risk of contamination across arms, though fidelity audits monitored and reported such events. Fourth, satisfaction was measured using an adapted instrument, which, despite validation, may not capture all dimensions of family-centered care. Finally, we did not include a cost-effectiveness analysis, which should be addressed in future studies.

## 5. Implications for emergency nurses

The implementation of personalized nursing interventions based on the MPEWS has been demonstrated to significantly enhance vital sign stability, shorten hospitalization duration, and reduce complications in acutely ill children. These findings support the integration of such scoring tools into the routine practice of emergency nurses to optimize patient monitoring and facilitate rapid intervention decision-making. To ensure effective utilization, emergency nurses should be subjected to additional specialized education, including dedicated training in MPEWS application, as well as structured training programs to enhance disaster nursing and emergency response capabilities.

### 5.1. Limitations

This study has several limitations. First, it was conducted in a single-center with a relatively small sample size, which may reduce the external validity of the findings and limit their generalizability to other healthcare settings or patient populations. Second, the retrospective cohort design may introduce potential selection bias and unmeasured confounding, despite the use of predefined inclusion criteria and standardized data extraction procedures. Third, the observation period was restricted to 72 hours, and longer-term outcomes such as readmission, neurological sequelae, or post-discharge mortality were not evaluated. Finally, parent satisfaction was assessed using a culturally adapted version of an existing questionnaire, which, although demonstrating good reliability, may not comprehensively reflect all aspects of family-centered care.

## 6. Conclusion

This study demonstrates that MPEWS-based personalized care significantly improves clinical outcomes for critically ill children in EOUs. The intervention reduced illness severity, stabilized vital signs more rapidly, shortened length of stay, decreased complications, and enhanced family satisfaction compared with routine care. These findings suggest that integrating MPEWS into standardized emergency care protocols may support earlier recognition of deterioration, guide timely multidisciplinary interventions, and provide a practical, scalable framework for improving pediatric emergency care quality and safety.

## Author contributions

**Conceptualization:** Yafei Liu, Xuejuan He, Lifeng Wei, Hui Hu, Yilin Miao, Lingfang Yu, Yuqian Weng.

**Data curation:** Yafei Liu, Xuejuan He, Lifeng Wei, Hui Hu, Yilin Miao, Lingfang Yu, Yuqian Weng.

**Formal analysis:** Yafei Liu, Xuejuan He, Lifeng Wei, Hui Hu, Yilin Miao, Lingfang Yu, Yuqian Weng.

**Investigation:** Lifeng Wei, Yilin Miao, Yuqian Weng.

**Funding acquisition:** Xuejuan He, Lifeng Wei, Hui Hu, Yilin Miao, Yuqian Weng.

**Writing** – **original draft:** Yafei Liu, Lingfang Yu, Yuqian Weng.

**Writing** – **review & editing:** Yafei Liu, Yilin Miao, Lingfang Yu, Yuqian Weng.
